# Longitudinal Pancreatojejunostomy for Pancreaticodigestive Reconstruction in the Resection of Pancreatic Head Malignancy with Chronic Pancreatitis: A Case Report

**DOI:** 10.70352/scrj.cr.24-0015

**Published:** 2025-03-04

**Authors:** Hironori Hayashi, Yuichiro Furutani, Hiroaki Sugita, Kei Sugano, Takahiro Yoshimura, Tetsuro Oda, Daisuke Fujimori, Koichiro Sawada, Masanori Kotake, Kaeko Oyama, Shintaro Yagi, Takuo Hara

**Affiliations:** 1 Department of Surgery, Kouseiren Takaoka Hospital, Eiraku-machi, Takaoka, Toyama, Japan; 2 Department of Hepato-biliary-pancreatic and Transplant Surgery, Kanazawa University Hospital, Takara-machi, Kanazawa, Ishikawa, Japan

**Keywords:** autoimmune pancreatitis, chronic pancreatitis, pancreatoduodenectomy, pancreatolithiasis, pancreatojejunostomy, whipple procedure

## Abstract

**INTRODUCTION:**

With progress in pancreatic surgery, a preservation of residual organ function has become more important. Pancreatic malignancies are occasionally accompanied by chronic pancreatitis (CP) and pancreatolithiasis (PL). Longitudinal pancreatojejunostomy (LPJ) is reportedly a useful method of surgical management in cases of CP with PL. We describe a patient with pancreatic head intraductal papillary mucinous carcinoma (IPMC) concomitant with PL, who underwent subtotal stomach-preserving pancreaticoduodenectomy (SSPPD) and LPJ for reconstruction.

**CASE PRESENTATION:**

A man in his 70s was referred to our hospital with a pancreatic head tumor. He had been treated for CP, diabetes mellitus, and chronic kidney disease. Imaging revealed a cystic pancreatic head tumor with a solid component that was histologically confirmed as IPMC. In addition, multiple calcifications suggestive of PL were observed in the pancreatic body and tail. SSPPD and LPJ were performed to excise the PL as much as possible and preserve the residual pancreatic function. The postoperative course was uneventful, and no abdominal symptoms or tumor recurrences were observed for approximately 8 months after surgery.

**CONCLUSION:**

This patient with IPMC with residual pancreatic PL was treated with SSPPD and LPJ to maximize the residual pancreatic function and reduce the occurrence of postoperative pancreatitis.

## Abbreviations

AIP
: autoimmune pancreatitis
CA19-9
: carbohydrate antigen 19-9
CEA
: carcinoembryonic antigen
CP
: chronic pancreatitis
DM
: diabetes mellitus
EPST
: endoscopic pancreatic sphincterotomy
ESWL
: extracorporeal shock wave lithotripsy
IPMC
: intraductal papillary mucinous carcinoma
LPJ
: longitudinal pancreaticojejunostomy
MPD
: main pancreatic duct
PD
: pancreatoduodenectomy
PL
: pancreatolithiasis
SSPPD
: subtotal stomach-preserving pancreaticoduodenectomy

## INTRODUCTION

Chronic pancreatitis (CP) is an inflammatory disease characterized by irreversible destruction of the pancreas with progressive loss of functional parenchyma, which results in endocrine and exocrine insufficiency.^1)^ CP is also a significant risk factor for pancreatic malignancies.^2)^ Due to recent progress in the treatment of pancreatic diseases, preservation of residual organ function has become more important in terms of better quality of life and perioperative multimodal management, similar to that observed with radicality. Longitudinal pancreaticojejunostomy (LPJ) is reportedly a useful surgical procedure as part of Frey’s surgery, with high safety in patients with CP.^3,4)^ However, to our knowledge, only one small case series has reported on the application of LPJ in the reconstruction of pancreatoduodenectomy (PD) in patients requiring resection for pancreatic head malignancy.^5)^

Herein, we describe a patient with pancreatic head intraductal papillary mucinous carcinoma (IPMC) concomitant with CP and pancreatolithiasis (PL) for whom we performed subtotal stomach-preserving PD (SSPPD) with LPJ for safe and efficient pancreaticodigestive anastomosis.

## CASE PRESENTATION

**Fig. 1:**
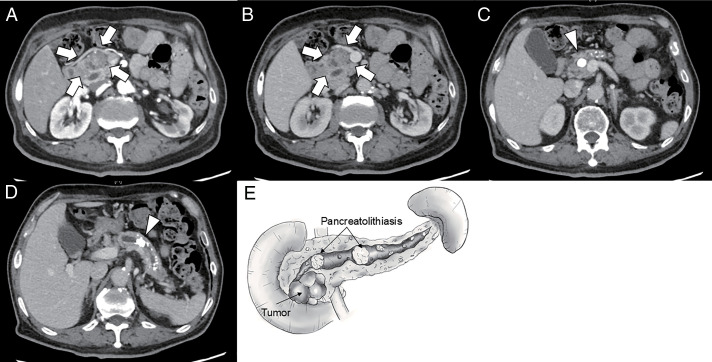
The pancreatic head tumor was identified as a hypoattenuating lesion in the early contrast phase (white arrow) (**A**). The tumor was also recognized as a progressively enhanced lesion over time (white arrow) (**B**). Multiple calcifications, suggesting pancreatolithiasis, were observed in the pancreatic parenchyma. In addition, high-density round lesions, approximately 2 cm in diameter, were observed in the dilated main pancreatic duct (white arrowhead) (**C, D**). Schema of the case (**E**).

A man in his 70s was referred to our hospital on his first visit with a suspected pancreatic head tumor. He had no history of drinking and smoking. The patient had been followed up at a nearby clinic for autoimmune pancreatitis (AIP) and was not taking any medication because of the patient’s poor compliance with treatment and lack of awareness of the disease. He had only accepted self-injected long-acting insulin treatment for diabetes mellitus (DM) (4 units per day). The patient also reported having occasional epigastric pain, which we diagnosed as a symptom of CP. Contrast-enhanced computed tomography revealed diffuse swelling of the pancreas and a cystic tumor with a solid component on the ventral side of the pancreatic head. The lesion was recognized as a hypoattenuating tumor in the early contrast phase (**Fig. 1A**). In addition, the tumor was recognized as a progressively enhanced lesion over time (**Fig. 1B**). Small cystic structures were also observed inside the tumor, indicating the possibility of IPMC (**Figs. 1A** and **1B**). Therefore, we performed an endoscopic ultrasound fine-needle aspiration biopsy for the pathological diagnosis of the lesion, which revealed a low-echogenic mass, with the specimen revealing IPMC. In addition, multiple calcifications suggestive of PL were scattered throughout the pancreatic head, body, and tail, with a large PL (approximately 2 cm in diameter) in the main pancreatic duct (MPD) of the pancreatic head and body, which was consistent with CP (**Figs. 1C–1E**). Blood examination indicated elevated IgG4 and HbA1c with hypoalbuminemia (IgG4 = 154 mg/dL, HbA1c = 7.1%, albumin = 2.5 g/dL, CEA = 3.8 ng/mL, CA19-9 = 21.6 U/mL), but no other significant abnormal findings were noted. Based on these examinations, he was diagnosed with mixed-type IPMC confined to the pancreas head (T2N0M0 Stage IB). We planned SSPPD for the resection of the IPMC. However, because of the large PL impaction in the MPD in the pancreas body, the possibility of residual pancreatitis owing to PL in the MPD could not be ignored considering postoperative safety. Thus, we planned SSPPD as follows. At the time of resection, division of the pancreas was planned at the pancreas neck with confirmation of a negative margin by frozen section diagnosis. After that, we planned a trial retrieval of the large PL at the pancreas body from the cutting edge. In case of failed PL retrieval, the MPD would be opened longitudinally for easy retrieval and possible preservation of the residual pancreas, and the LPJ technique would be applied for safe reconstruction.

No liver or peritoneal metastases were detected intraoperatively. The bile duct was transected at the common hepatic duct, which crossed the right hepatic artery, and the pancreas was separated on the ventral side of the superior mesenteric vein using regional lymph node dissection. Intraoperative rapid pathological diagnosis did not confirm tumor cells or epithelial dysplasia at the resection margins. After the rapid pathological diagnosis, we tried to retrieve the PL from the cutting edge of the MPD. With failure despite repeated attempts, we decided to reconstruct the MPD using LPJ.

**Fig. 2:**
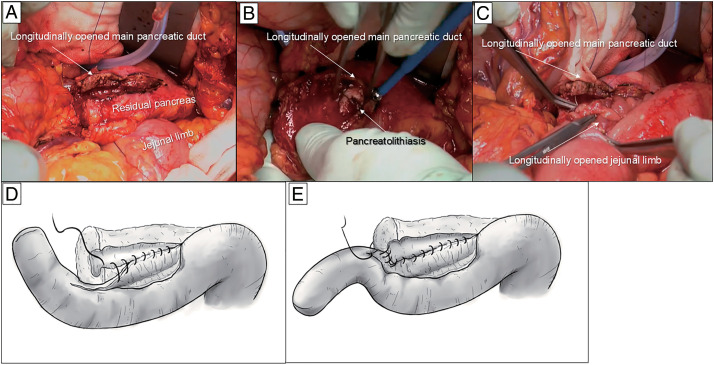
Intraoperative findings of LPJ. The main pancreatic ductotomy was performed, which opened longitudinally leftward as far as possible (**A**). The pancreatolithiasis was resected as far as possible before reconstruction (**B**). Side-to-side LPJ was performed using a 4-0 absorbable monofilament suture (**C, D**). Transpancreatic/jejunal seromuscular sutures were used to completely cover the pancreatic stump with jejunal serosa (**E**). LPJ, longitudinal pancreatojejunostomy

**Fig. 3:**
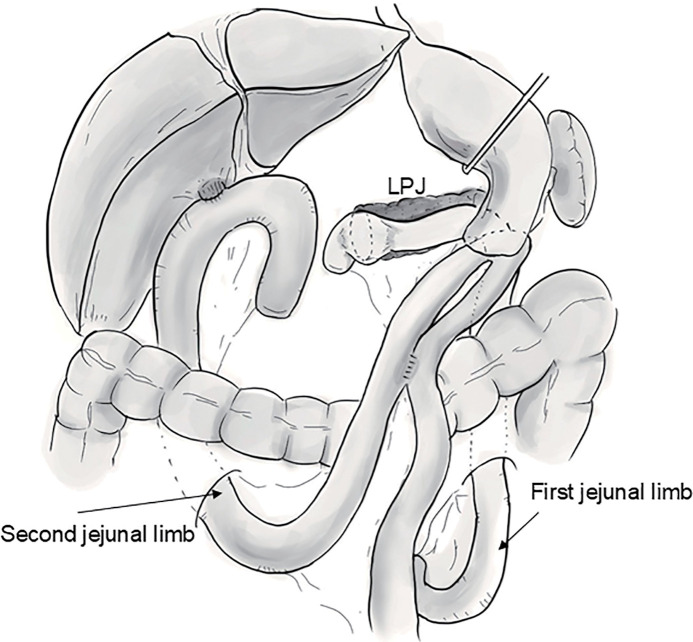
The schematic diagram of the reconstruction performed on this patient. The first and second Roux-en-Y limbs were elevated separately. Anastomosis of the first and second jejunal limbs was performed approximately 15 cm on the anal side of the Braun anastomosis.

**Fig. 4:**
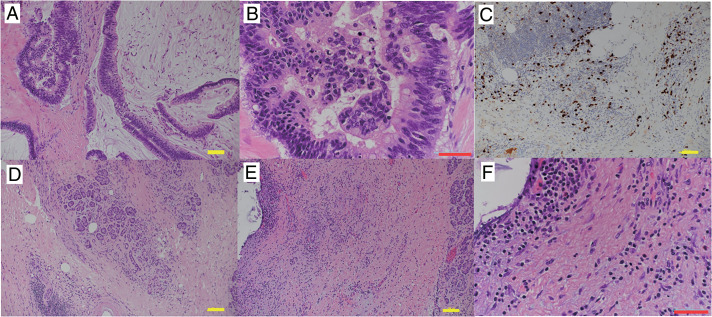
Histopathological examination revealed that the tumor was composed of mucinous carcinoma with well-differentiated tubular adenocarcinoma (**A, B**). In the non-tumorous pancreas, IgG4-positive cells were observed by immunohistochemical study (**C**). Moreover, moderate infiltration of plasma cells and lymphocytes was observed, but storiform fibrosis and obliterative phlebitis were unnoted (**D–F**) (scale bar in **A, C, D, E**: 100 μm; scale bar in **B**, **F**: 50 μm).

During reconstruction, we planned to divert the pancreaticojejunostomy and hepaticojejunostomy limbs to avoid contamination of the pancreaticojejunostomy site with bile. Details of the surgical reconstruction technique have been reported by Sudo et al.^6)^ The MPD of the residual pancreas and the PL were identified using intraoperative ultrasonography, and ductotomy was performed using electrocautery that opened longitudinally leftward as far as possible (**Fig. 2A**). The PL was removed as far as possible, and we carefully confirmed there were no neoplastic lesions in the residual pancreas using intraoperative ultrasonography before the reconstruction (**Fig. 2B**). After the first Roux-en-Y limb was created, the jejunal limb was opened on the opposite side of the mesentery, matching the length of the opening of the MPD. A side-to-side LPJ was performed using a 4-0 absorbable monofilament running suture in a single-layer fashion (**Figs. 2C** and **2D**). We used transpancreatic/jejunal seromuscular sutures to completely cover the pancreatic parenchyma to the jejunal seromuscular layer^7)^ (**Fig. 2E**). Subsequently, the second Roux-en-Y limb was elevated, and hepaticojejunostomy, gastrojejunostomy, and Braun anastomosis were performed. Finally, the first and second jejunal limbs were anastomosed, approximately 15 cm on the anal side of the Braun anastomosis (**Fig. 3**). The operative time was 595 min, with blood loss of 500 mL. Histopathological examination revealed that the tumor was composed of mucinous carcinoma with well-differentiated tubular adenocarcinoma (mixed-type invasive IPMC) with intraductal papillary mucinous neoplasm epithelium expansion to the MPD (**Figs. 4A** and **4B**). In the non-tumorous pancreas, IgG4-positive cells were observed by immunohistochemical study, with moderate infiltration of plasma cells and lymphocytes, loss of the pancreatic parenchyma, and fibrosis (**Figs. 4C** and **4D**). However, storiform fibrosis and obliterative phlebitis were unnoted (**Figs. 4E** and **4F**). Therefore, the background pancreas was diagnosed as having CP according to the Japanese diagnostic criteria.^8)^ However, AIP was not diagnosed histologically.^9)^ The histopathological diagnosis was T2N0M0 Stage IB, and R0 resection was achieved.

The patient’s postoperative course was uneventful, and he was discharged 27 days after surgery with self-injectable long-acting insulin (4 units/day) for DM and pancrelipase 900 mg per day for replacement therapy for pancreatic exocrine insufficiency. He has undergone 8 months of postoperative follow-up without any abdominal symptoms or tumor recurrence.

## DISCUSSION

CP causes irreversible pancreatic destruction with progressive loss of functional parenchyma, resulting in endocrine and exocrine insufficiency.^1)^ It is also a significant risk factor for pancreatic malignancies.^2)^ Therefore, surgical management of pancreatic malignancies concomitant with CP is important for better quality of life and perioperative multimodal management, similar to that achieved with radicality.

LPJ is reportedly an effective surgical technique with high safety in patients with CP.^3,4)^ However, to our knowledge, only one small case series (n = 8) on the application of LPJ in the reconstruction of PD in patients requiring pancreatic head malignancy resection has been published.^5)^ We safely performed surgical treatment of pancreatic head IPMC, concomitant with CP and PL, using SSPPD with LPJ.

During typical PD, reconstruction of the pancreatic stump is performed using the cut end of the pancreas; this is a challenging step with a significant risk of morbidity related to postoperative pancreatic fistulas.^10,11)^ During the reconstruction of the pancreatic stump, the cut end of the pancreas is usually mobilized at least 2–3 cm away from the surrounding tissues. Mobilization of the pancreatic stump may not be possible or may be inadequate because of technical difficulties, including pancreatitis, as in our case. In addition, retrieval of trapped PL in the MPD needs an unusual reconstruction technique due to the longitudinal opening to the side of the pancreas tail. Therefore, an alternative method of pancreaticojejunostomy, such as LPJ, should be considered for the safe reconstruction of this case.^5)^

In cases of pancreatic head malignancy with a large PL in the pancreatic body, as in the present patient, PL impacted in the MPD should be removed as much as possible. Thus, extended pancreatic resection including that of the pancreatic body might also be considered. However, we did not select extended resection, including that of the pancreatic body, for the following reasons: First, as mentioned above, extended resection of the pancreatic body is expected to be technically difficult in terms of pancreatic mobilization due to inflammatory changes in the surrounding tissue.^5)^ Second, extended resection of the pancreas, including the pancreatic body, is strongly associated with an insufficient residual pancreatic volume.^12)^ To preserve residual pancreatic function, residual pancreatic volume should be preserved as far as possible. Preservation of residual pancreatic function affects postoperative quality of life.^13)^ Thus, we consider that the LPJ preserved organ function as far as possible.

From a technical perspective, LPJ is not very complicated because of the wide-open anastomotic orifice, which is observed in a wide surgical field. A previous report recommended the creation of a large anastomotic orifice toward the pancreatic tail side.^6)^ In the present case, we followed this recommendation, which allowed easy suturing and good visualization of the anastomotic site.

However, LPJ should be cautiously performed in case of dysplastic epithelium at the cutting edge or expansion to the residual pancreas. There have been reports regarding the indication for additional resection of high-grade dysplastic epithelium at the cutting edge.^14,15)^ We planned additional resection of the residual pancreas in case of high-grade dysplasia at the cutting edge for the present case to completely resect the high-grade dysplastic epithelium. After pathological confirmation, PL retrieval in the MPD was tried from the cutting edge. The extent of dysplastic epithelium and indication for additional resection vary depending on the patient’s condition, including the background condition of the pancreas.

In patients with CP and PL, extracorporeal shock wave lithotripsy (ESWL) is reportedly an effective therapy for the extirpation of the PL without surgical intervention for symptomatic PL.^16,17)^ We were concerned that surgery would cause remnant pancreatitis and worsen his symptoms under body PL residuum. Therefore, we also considered preoperative ESWL for the present patient to facilitate PL removal in the MPD at the residual pancreas body. However, we did not apply it for the following reasons: First, ESWL for PL requires a specific therapeutic period. In this case, neoadjuvant chemotherapy was not administered because of the patient’s comorbidities, and radical surgery was performed as promptly as possible. Second, multiple PLs were evident, and the largest PL was approximately 2 cm in diameter. Thus, there was a risk of acute pancreatitis occurring due to the destruction of PL particles using ESWL. Additional endoscopic procedures, such as endoscopic pancreatic sphincterotomy (EPST), are needed to extirpate the PL and its particles after ESWL.^18)^ Such endoscopic interventions also have the risk of adverse events, which increase the risk of delayed pancreatectomy. Therefore, we selected upfront SSPPD with LPJ reconstruction as our therapeutic strategy.

During the reconstruction, we chose to separate the LPJ and choledocojejunostomy loops. Pancreatic juice is activated through the mixing of bile components,^19)^ and activated pancreatic juice may adversely affect the remnant pancreas. Thus, the anastomotic loops were separated, and such possibilities were excluded. No adverse events, such as remnant pancreatitis, anastomotic leakage, or pancreatic fistula, were observed. However, there are controversies regarding the dual limb in PD reconstruction.^20)^ Regarding the integrity of pancreatojejunostomy, a recent study reported the absence of a significant difference in PJ anastomosis with prolonged operation time. Thus, a general indication for dual limbs should be cautiously determined because of the absence of significant benefits.

The present case needed careful follow-up with consideration for de novo pancreatic invasive ducatal adenocarcinoma (PDAC) from CP, even postoperatively. The increased risk of PDAC and increased incident rate of PDAC with CP have been reported.^21)^ However, there is no standard follow-up program for managing PDAC occurrence in CP. Our follow-up plan included monthly tumor marker tests and imaging studies for at least 6 months in this case. It is desirable to determine a follow-up method for PDAC in patients with CP.

This study had some limitations. First, the patient’s preoperative and postoperative pancreatic endocrine and exocrine function were not evaluated. His pancreatic function had clearly deteriorated due to the CP. However, the patient had not experienced any other symptoms except for DM. Therefore, he refused to undergo further examination of pancreatic function. Second, from an oncological perspective, this patient should not have been treated with surgery alone, but with adjuvant chemotherapy to improve his prognosis.^22)^ We informed the patient and his family of the significance of the adjuvant chemotherapy. However, the patient did not wish to undergo adjuvant therapy.

## CONCLUSION

This patient with IPMC and residual PL was treated using SSPPD with LPJ to maximize his residual pancreatic function and reduce the risk of occurrence of postoperative pancreatitis. LPJ is a safe and effective surgical procedure for the treatment of PL with CP that maximizes residual pancreatic preservation.

## ACKNOWLEDGMENTS

The authors thank Dr. Isamu Makino (Kanazawa University Hospital) for his helpful assistance with the surgical management, Dr. Kazuhiro Nomoto (Department of Pathology, Kouseiren Takaoka Hospital) for his assistance with the pathological diagnosis, and Dr. Takaaki Wada (Ichinose-Wada Clinic) for clinical management of the patient.

## DECLARATIONS

### Funding

This study was not funded by any grant.

### Authors’ contributions

HH performed the surgical management and treatment, analyzed the case, and drafted the manuscript.

YF, HS, DF, and KSa participated in the surgical treatment and patient care with clinical data collection.

KSu created the schema and analyzed the literature. TY, TO, and KO analyzed the literature.

MK, SY, and TH contributed to the study design and participated in the surgical treatment planning.

All the authors have read and approved the final version of this manuscript.

### Availability of data and materials

All data generated or analyzed during this study are included in the published article.

### Ethics approval and consent to participate

Not applicable.

### Consent for publication

Written informed consent was obtained from the patient and his family for the publication of this paper and the accompanying images.

### Competing interests

The authors declare that they have no competing interests.
